# ReacKnock: Identifying Reaction Deletion Strategies for Microbial Strain Optimization Based on Genome-Scale Metabolic Network

**DOI:** 10.1371/journal.pone.0072150

**Published:** 2013-12-11

**Authors:** Zixiang Xu, Ping Zheng, Jibin Sun, Yanhe Ma

**Affiliations:** 1 Key Laboratory of Systems Microbial Biotechnology, Chinese Academy of Sciences, Tianjin, China; 2 Tianjin Institute of Industrial Biotechnology, Chinese Academy of Sciences, Tianjin, China; Semmelweis University, Hungary

## Abstract

Gene knockout has been used as a common strategy to improve microbial strains for producing chemicals. Several algorithms are available to predict the target reactions to be deleted. Most of them apply mixed integer bi-level linear programming (MIBLP) based on metabolic networks, and use duality theory to transform bi-level optimization problem of large-scale MIBLP to single-level programming. However, the validity of the transformation was not proved. Solution of MIBLP depends on the structure of inner problem. If the inner problem is continuous, Karush-Kuhn-Tucker (KKT) method can be used to reformulate the MIBLP to a single-level one. We adopt KKT technique in our algorithm ReacKnock to attack the intractable problem of the solution of MIBLP, demonstrated with the genome-scale metabolic network model of *E. coli* for producing various chemicals such as succinate, ethanol, threonine and etc. Compared to the previous methods, our algorithm is fast, stable and reliable to find the optimal solutions for all the chemical products tested, and able to provide all the alternative deletion strategies which lead to the same industrial objective.

## Introduction

In the 21st century, metabolic engineering has been pinned outstanding hopes on many aspects, such as energy, environment pollution, climate improvement, food sources and human health [1]. Since Jay Bailey, Joe Valino and Greg Stephanopoulos published their classic papers [2], [3] of metabolic engineering, the concepts and methods of metabolic engineering have been elucidated in the 1990s. The technique of DNA recombinant made it possible to manipulate genetic changes, and this effectively broke through the traditional breeding possibilities and was widely applied to industrial production strains. As one of the main technologies of recombinant DNA, gene knockout method was widely used to improve the conversion ratio of strains for the products. Earlier gene deletion strategies were mainly based on the analysis of local metabolic pathway and the experience of experiment. With the development of systems biology and synthetic biology, utilizing cellular network model combining with different mathematical methods, genetic operation in metabolic engineering tended to rationality and metabolic engineering has come into the era of system metabolic engineering [4]. At the same time, constraint-based modeling (CBM), including genome-scale metabolic network models [5]–[12] and flux balance analysis (FBA) [13], made it possible to bypass the requirements of detailed enzyme kinetic information by analyzing the function of genome-scale metabolic networks through relying solely on simple physical–chemical constraints.

There were a series of published algorithms to predict the target reactions for deletion to improve the productivity of chemicals. OptKnock [14] used bi-level optimization strategy to solve the conflict of cell growth and maximum bioengineering objective; RobustKnock [15] was similar to OptKnock, but utilized min-max strategy to get a more robust solution; the GDLS algorithm [16] was used for reduced metabolic models employing Gene-Protein-Reaction associations to predict gene knockouts; OptGene [17] used evolutionary search procedure for solving the resulting combinatorial optimization problem; OptReg [18] and OptStrain [19] extended OptKnock in some functions as fusing non-host reactions and the strength of gene expression. Main characteristics of these algorithms were: 1) based on metabolic networks, 2) towards reactions as deletion targets, 3) bi-level strategy, exactly mixed integer bi-level linear programming (MIBLP). When solving the bi-level optimization, these algorithms learnt from OptKnock, used duality theory and transformed bi-level optimization to single-level one.

The motivation of this study is: Although it is a good idea to use bi-level optimization strategy to coordinate cellular growth and bioengineering objective, where the MIBLP contains integer control variables of the upper problem appearing in the inner problem, the solution of large scale MIBLP is intractable and there are only a few methods [19], as we know, which can be used to solve it. OptKnock was the base of other algorithms (RobustKnock, GDLS and so on) and OptKnock just cited a previous work [20] where the authors did not prove the validity of their solving method for MIBLP as well. The method used in OptKnock and so on was to regard the control variables of the upper problem as parameters, to transform the inner problem to its dual form, to require the primal and dual objectives to be equal and then to combine them, and finally got a single level one, a mixed integer linear programming. However, they did not include the lower-level primal variables in the dual objective and thus, erroneously derived a MILP (mixed integer linear programming) formulation, as also stated by DeNegre in his dissertation (page 15) [21]. Explicitly, in the process of transforming to single level problem and when assigning auxiliary variables to inner problem, the constraint 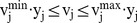 was not assigned. If it was assigned, a mixed integer nonlinear programming (MINP) would be derived, but not a MILP one. We provided an appendix as **Text S1** to point out the problem.

The solving approaches for the MIBLP problems depend only on the structure of inner problem [21]. Although it is difficult to cope with the large-scale MIBLP with integer control variables of the upper problem but appearing in the inner problem, if the inner problem is continuous, Karush-Kuhn-Tucker (KKT) method can be used to reformulate the MIBLP to a single level one [22], [23]. In this study, we adopt KKT technique [24], [25] to attack the intractable problem of the solution of MIBLP. We defined the name of our algorithm as ReacKnock, for the targets that our method predicted were reactions as well.

## Methods

### Mathematical presentation of ReacKnock

The mathematical model of ReacKnock was similar to OptKnock and it was also a bi-level optimization structure. The first level (the upper problem) was to maximize bioengineering objective (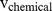) and the second level (the inner problem) was for cellular growth (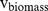). But we made a small modification to the model of OptKnock for concision. We moved the constraints of 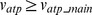 and 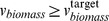 to be included in 
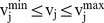
. Binary variable *y* vector was used to indicate some reactions being deleted or not. The mathematical expression of ReacKnock was:
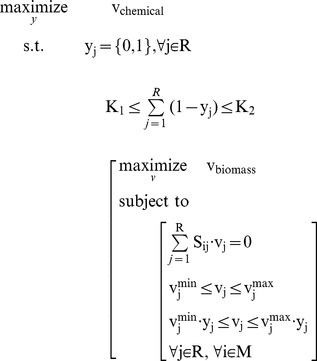
(I)Here M is the set of metabolites and R is the set of reactions with size r; 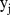 is the control variable of the j-th reaction of R, and it will force the flux 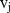 to zero in case of 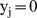, mimicking the gene knockout scenario; 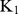 and 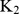 are the scope for search; S is the stoichiometry matrix, *v* is the distribution of flux; 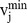 and 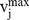 are the flux boundaries of every reaction.

### Method to solve the MIBLP model

For the solution of bi-level linear programming (BLP), KKT method can be used to transform bi-level problem to a single level problem [22], [23]. Audet and Bard have given the transformation [24], [25]. Firstly, we reformulated the MIBLP to a standard formation
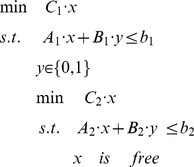
(II)Here y is the control variables from upper level, *x* is corresponding to flux v. A_1_, B_1_, C_1_, A_2_, B_2_, C_2_ are matrixes in proper dimensions.
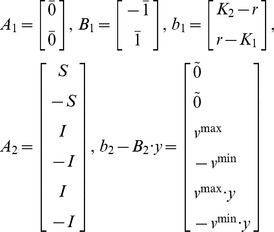
Here 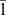 is 1×r vector with element 1, 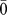 is 1×r vector with element 0 and 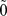 is r×1 vector with element 0.

The Lagrangian for the inner is 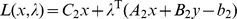. The KKT condition for the standard inner problem can be derived as the following.
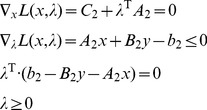
(III)Where 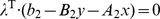 is equivalent to 
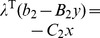
, which can be further written as
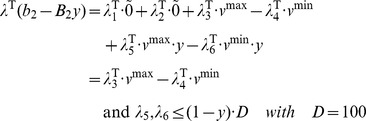
The nonlinear terms 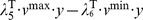 can be removed. This is due to if y = 0, they will be zero; or if y = 1, the corresponding constraints for the auxiliary variables 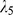 and 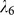 are repeated constraints and thus inactive, the auxiliary variables 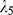 and 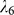 will be zero.

So the above MIBLP (II) can be reformulated to a single level one, a MILP (IV) through (III).
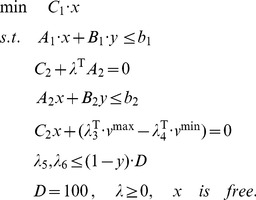
(IV)This MILP can be solved by some commercial softwares, such as Gurobi 5.0 [26].

### Alternative solution

The above MILP (IV) may probably have multiple integer solutions, i.e. for different deletion strategies but the industrial objective was the same. As we knew, up to date, there was no optimization tool which can directly provide multi integer solutions for a MILP. Here we utilized an approach named Combinatorial Bender's cut [27] to get those alternative integer solutions. The idea of Bender's cut proposed by Balas and Jeroslow was that from an existing solution, iteration was used while the following binary cut was added in each iteration to exclude an existed solution.
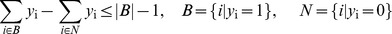
All the multiple solutions can be obtained by this way.

### Method to testify the deletion strategies

When we obtain a deletion strategy from a prediction algorithm, we get the values of cell growth and industrial objective at the same time. It is best to substitute the deletion strategy to the metabolic network model, delete those target reactions (or enzymes) predicted, do the FBA, and see whether cell growth and industrial objective are the same with the values we predict by our algorithm. But for the reason that FBA usually has multi solutions, so when we do FBA testification and if a strategy does not get to the predicted value of industrial objective, we can't decide the strategy is valid or not. But FBA can be used to testify growth.

To multiple solutions of FBA, Flux Variability Analysis (FVA) can provide an estimation of the flux scope of every reaction in the FBA model. We think it is a good way to testify deletion strategies predicted. After substituting the deletion strategy to the metabolic network model, delete those target reactions (or enzymes) predicted, we do the FVA now and compare the maximum value of industrial objective with what we predict.

## Results

To evaluate the performance of ReacKnock in comparison with previous algorithms (OptKnock), we applied ReacKnock on a genome-scale metabolic network model of *E. coli* metabolism, named iAF1260 [5], to predict knockout strategies for producing various chemicals. Predicted strategies provided were 5 reaction deletions. The metabolic network model includes 1260 enzyme-coding genes, accounting for 2382 reactions and 1668 metabolites. Focusing on minimal medium with glucose as sole carbon source, we applied ReacKnock and OptKnock respectively towards the production of different chemicals that can be secreted from *E. coli*. We can't obtain the original algorithm program of OptKnock and OptKnock algorithm in this study was from the corresponding function of COBRAToolbox [28], but it was not clear whether the OptKnock function in COBRA has been modified and been corrected from the original paper [14]. **Table 1** gave the comparison result under aerobic condition. Organic acids producible from *E. coli* may be produced probably both under anaerobic condition (such as Ethanol) and aerobic condition (such as Threonine). Here, it was for the reason of computational aspect that we used unified aerobic condition. Of course, it is easy to get the results under anaerobic condition. In order to show the change of flux distribution after removing those target reaction, intracellular flux distributions for each chemical production using ReacKnock and OptKnock were respectively provided in **Table S1 and S2**, and flux distribution of wild strain was also provided in the tables for comparison. At the same time, detailed maps of metabolic flux distributions were respectively provided in **Figure S1** where those reactions with relatively large flux were shown. As for the whole names of Knockout enzymes (reactions), please refer to the supplementary materials of Ref [5] where it provided the whole names of every reaction. The software to solve MILP that we used here was Gurobi 5.0 and Matlab [29].

**Table 1:** Comparison of the predictions by ReacKnock, Optknock and Wild_type.

Chemical target	Strain	Prod. rate	Growth rate	FVA max Prod. rate	FBA max Growth rate	Reactions to be deleted as example
Succinate	(Max_Yield)	14.93				
	Wild_type	0	0.885	0.0001		–
	ReacKnock	9.96	0.1173	9.96	0.1173	ACtex; ATPS4rpp; CO2tex; PGL; THD2pp
	OptKnock	6.3	0.552	6.31	0.552	ACt2rpp; GND; PSP_L; SUCDi; SUCOAS
Ethanol	(Max_Yield)	18.56				
	Wild_type	0	0.885	0.000024		–
	ReacKnock	18.5	0.104	18.46	0.104	ATPS4rpp; FORtex; GLUDy; O2tex; THD2pp
	OptKnock	18.2	0.121	18.19	0.121	ACt2rpp; ATPS4rpp; GLUDy; PPKr; SUCDi
Acetate	(Max_Yield)	25.69				
	Wild_type	1.68	0.885	1.68		–
	ReacKnock	22.7	0.145	22.7	0.145	CO2tex; F6PA; GLCDpp; PFK; PGL
	OptKnock	18.3	0.116	18.25	0.116	3OAS120; ATPS4rpp; ENO; GLU5K; SUCDi
Hydrogen	(Max_Yield)	76.64				
	Wild_type	9.81	0.885	9.81		–
	ReacKnock	66.65	0.119	66.65	0.119	ETOHt2rpp; G6PDH2r; H2Otpp; PGM; TKT2
	OptKnock	1000.0	0.885	9.81	0.885	no deletion
Formate	(Max_Yield)	43.69				
	Wild_type	0.0021	0.885	0.00223		–
	ReacKnock	32.08	0.127	32.08	0.127	ACALD; EDD; ENO; H2Otex; PPS
	OptKnock	25.5	0.142	25.54	0.142	12PPDStex; H2Otex; PGI; PGL; PGM
Glycolate	(Max_Yield)	25.69				
	Wild_type	0	0.885	0.000039		–
	ReacKnock	18.27	0.129	18.27	0.129	ACtex; AKGDH; ATPS4rpp; MALS; PGCD
	OptKnock	17.4	0.142	17.43	0.142	ACtex; AKGDH; ATPS4rpp; FALDtpp; GLCNtex
D-Lactate	(Max_Yield)	18.56				
	Wild_type	0	0.885	0.000019		–
	ReacKnock	18.52	0.10	18.52	0.10	ASNS2; ATPS4rpp; CBMKr; ETOHtex; O2tex
	OptKnock	18.5	0.101	18.51	0.101	ATPS4rpp; ETOHt2rpp; IMPD; LEUtex; O2tex
Fumarate	(Max_Yield)	16.08				
	Wild_type	0	0.885	0.0000082		–
	ReacKnock	13.45	0.152	13.45	0.152	CO2tex; CYTBO3_4pp; FORtppi; PDH; PYRtex
	OptKnock	9.4	0.127	9.39	0.127	3HAD140; ATPS4rpp; CO2tpp; PFL; TKT2
Threonine	(Max_Yield)	11.22				
	Wild_type	0	0.885	0		–
	ReacKnock	0	0.885	0	0.885	ANHMK;DHORD5;GTHRDHpp;OMBZLM;VPAMT
	OptKnock	0.000000012	0.774	0.0000237	0.774	ACALDtpp; ACtex; ETOHt2rpp; Htex; TRPS1

The following constraints were applied: glucose consumption rate is 10, cell growth is no less than 0.1, maintenance energy metabolism is 8.39, oxygen consumption rate is no higher than 18.5. All the rate unit is mmol/g(Dw)h. Max_yeild means the maximum conversion ratio at the given condition.

There are several merits of our algorithm over previous methods. 1) First and especially, ReacKnock will return all the alternative deletion strategies in the same search scope with the near industrial objective. This will be very useful in strain design and can provide alternative gene operation strategies. All the previous algorithms just give only one deletion strategy for a given deletion number. Table 2 has shown the first ten alternative solutions for predicting 6-reaction deletions to produce Succinate on the *E. coli*_iAF1260. All these ten solutions were consistent with the results of FVA and FBA. Intracellular flux distributions for each solution were provided in **Table S3**. 2) In most cases, the objective value for a given chemical predicted by ReacKnock is higher than the value predicted by OptKnock and is much more near the theoretical conversion ratio (Max_yield). As we demonstrated in Appendix, the solving method of OptKnock for MIBLP was not precise in mathematics, and we think this may be the first reason why OptKnock is unable to find the optimal solution. When running OptKnock for these chemical targets, we have set the maximum computation time to be 3600 s. The second reason may lie in that it will take a very long time to get the optimal solution while the permitted time is not enough. We have also tried not to set the maximum computation time (actually default setting in COBRA toolbox) and the yields of the targeted chemical products seemed not improved obviously. We provided new solutions and computation time of OptKnock in **Table S4**. Although there was a distance between the maximum production predicted by ReacKnock and the theoretical conversion ratio, this is due to the constraint of deletion number. As an example, we computed 15-reaction deletion for succinate production which could get the maximum production to 13.97. The 15 reactions were “3OAS120, AKGt2rpp, CO2tpp, FUMtex, GLCNt2rpp, GLUDy, GLYCL, GND, PDH, PFL, PGI, PPKr, PSP_L, PYRt2rpp, TDECOAI”. 3) ReacKnock is stable and all the rates of chemical objective predicted by ReacKnock were consistent with the results of FVA and FBA. OptKnock is instable in the cases of Hydrogen production, and it can't provide effective deletion strategies for the chemical productions. 4) Our algorithm does not confine to a given number of gene deletions and it searches in a given scope defined by K1 and K2, such as between 5 to 20 genes. 5) Our algorithm will need shorter time than previous approaches. Our computation environment is a server, sugon A840-G with 4 AMD Opterons and 48 cores, and CPU speed is 2.2 GHz. When using OptKnock to do 5 gene deletion study, it will need tens of minutes in a run, and of course, will need much more in actual gene deletion calculations (such as >10 genes). In general, our algorithm will need no more than several minutes in a run and especially do not restrict to the scale of gene deletion. All the computational results of ReacKnock were obtained with setting computation time as 10 minutes. Actually, OptKnock will need more time for we have set the terminal time to be 3600 s when running it, and for comparison and in **Table S4**, we also provided the result of OptKnock with setting computation time as 10 minutes.

**Table 2:** First ten alternative solutions provided by ReacKnock for predicting 6-reaction deletions to produce succinate on the model *E. coli*_iAF1260 under aerobic condition with glucose Input = −10 mmol/g(Dw)h.

1	2	3	4	5	6	7	8	9	10
ACt2rpp	ATPS4rpp	ACGAMK	ACtex	3HAD140	ACtex	ACtex	ACtex	ACtex	ACtex
ATPS4rpp	CBMKr	ACt2rpp	ATPS4rpp	ATPS4rpp	ATPS4rpp	ATPS4rpp	ATPS4rpp	ATPS4rpp	ATPS4rpp
CO2tpp	CBPS	ATPS4rpp	CO2tex	CO2tpp	CO2tex	CO2tex	CO2tpp	CO2tex	CO2tpp
PGL	CO2tpp	IDOND	G6PDH2r	FORtex	GND	PSERT	G6PDH2r	GND	G6PDH2r
PSERT	PFL	PSP_L	PSP_L	FUM	PSERT	THD2pp	PGCD	PSP_L	PSP_L
THD2pp	THD2pp	SUCDi	THD2pp	RPE	THD2pp	TKT1	THD2pp	THD2pp	THD2pp
0.108	0.109	0.129	0.108	0.128	0.108	0.108	0.108	0.108	0.108
10.24	9.73	9.13	10.24	9.35	10.24	10.20	10.24	10.24	10.24

The last two lines are growth rate and product rate respectively.

## Discussion

For the scarcity of dynamic data, genome scale metabolic network models based on constraint-based modeling (CBM) provide a possible way to describe the metabolism of cells. FBA has been used successfully to simulate the phenotype of cells. But in metabolic engineering, we hope to know *in silicon* what the response of cell to the gene deletion operation is. Therefore, bi-level optimization is a pertinent strategy to consider cell growth and industrial objective together. But when we use integer control variables to indicate the decision of deletion or not for some reactions, this type of bi-level optimization, known as MIBLP, become very difficult to solve. In fact, the inner problem of MIBLP on which we focus here is continuous and the solving approaches for MIBLP depend only on the structure of inner problem. In this article, we utilize KKT method to solve the MIBLP model which is the core of previous predictive algorithms of gene deletion study.

There are several merits of ReacKnock: 1) Our algorithm can provide all the alternative deletion strategies in given deletion number. 2) ReacKnock may give better predictions than previous methods in term of production rate or conversion ratio. 3) ReacKnock is more stable and reliable than previous tools. ReacKnock may obey the original FBA model, i.e. applying FVA to predict the possible production rate under knockout strategies of ReacKnock reveals possible rates that are very near to the results of ReacKnock. 4) The computation time of ReacKnock is greatly shorter than that of previous algorithms. 5) ReacKnock does not confine to single, double or triple knockouts, and it searches a scope which we can define at first, such as 5–20 reactions (enzymes). ReacKnock will return the best set which should be deleted.

It should be noticed that, sometimes, the optimal knockout strategy found by ReacKnock and OptKnock is not SUFFICIENT condition for optimal production. Due to the inherency of multiple solutions of FBA approach, ReacKnock and similar can only ensure that there is at least a distribution of metabolic flux leading to maximal production yield. This means with the given set of gene deletions, there are multiple flux distribution modes which all have the same maximal growth rate but different production rate. Only a few modes lead to the maximal production rate. Therefore, the experimental practice according to the prediction of ReacKnock or OptKnock may show less optimal or even bad production yield. It is of strong need to develop new algorithms searching for sufficient solutions with which the cell is obligated to produce the target product at the maximum yield.

Alternative deletion strategies are actually interrogation of multiple solutions of the MILP (mixed integer linear programming) transformed from MIBLP. COBRA toolbox provides a method named random sampling [28], [30], [31] to determine the size and shape of the steady-state flux space defined by the constraint–based model of metabolic network, i.e. a linear optimization problem (LP). The random sampling in COBRA toolbox is Monte Carlo sampling. Solution space (usually a polytope for a LP) is different from alternative solutions of the LP (usually vertexes of the polytope for the LP), if the LP has multiple solutions. Vertex enumeration will be helpful to calculate the volume of the LP, but is usually N-P hard problem. But in our case, the optimization problem is a MILP transformed from the MIBLP. Multiple solutions of those integer variables for the MILP are of discrete problem, and the discrete property determines that it is not suitable for sampling. At the same time, multiple solutions of our MILP are usually just several or tens but not a great many as tested, and we hope to get these solutions one by one by the method of Bender's cut. So we believe Bender's cut is suitable for our MILP model solution to interrogate alternative deletion strategies. Iterative method has been utilized before for finding multiple solutions of a LP [32], [33], supposing that the LP has multiple solutions. This iterative method for LP is a little different from the Bender's cut method used here for MILP.

## Supporting Information

Figure S1 **Maps to show the intracellular flux distributions for each chemical production after deleting those target reactions predicted by ReacKnock and OptKnock.** The intracellular flux distribution of wild strain was also provided for comparison.
(RAR)

Text S1 **Appendix to point out where the problem of OptKnock is and to give the derivation of our algorithm.**
(DOCX)

Table S1 **Intracellular flux distributions for each chemical production after deleting those target reactions predicted by ReacKnock.**
(XLSX)

Table S2 **Intracellular flux distributions for each chemical production after deleting those target reactions predicted by OptKnock.**
(XLSX)

Table S3 **Intracellular flux distributions of multiple strategies for succinate production after deleting those target reactions by using ReacKnock.**
(XLSX)

Table S4 **Comparison between new predictions of OptKnock with default setting of max computation time in COBRA toolbox and old predictions (first time computation) of OptKnock with setting computation time to be 3600 s.**
(DOCX)
