# Supplementary material for: ReacKnock: Identifying Reaction Deletion Strategies for Microbial Strain Optimization Based on Genome-Scale Metabolic Network
Source: PLoS One. 2013 Dec 11;8(12):e72150. doi: 10.1371/journal.pone.0072150 (PMC3859475; doi:10.1371/journal.pone.0072150)
Supplement: Text S1 — Appendix to point out where the problem of OptKnock is and to give the derivation of our algorithm. (DOCX) [file pone.0072150.s002.docx]

**Appendix: Where is the problem of OptKnock ?**

The method used in OptKnock for solving MIBLP was to regard the control variables *y* of the upper problem as parameters and to transform the inner problem to its dual form, which requires the primal and dual objectives to be equal and then to combine them as a constraint, and finally got a single level one, a mixed integer linear programming (MIP).

The inner problem of mixed integer bi-level linear programming (MIBLP) in OptKnock was as the following (I) (from the paper of OptKnock)

(I)

Its corresponding dual was as the following (II) (from the paper of OptKnock)

(II)

When transforming the inner problem (I) to its dual form (II), auxiliary variables were assigned to those constraints. As stated in the paper of OptKnock, $\lambda_{\text{i}}^{\text{stoich}}$was the dual variable associated with i-th stoichiometric constraint ($i\in N$, N was the set of all the compounds), *glc* was the dual variable associated with the glucose uptake constraint $v_{pts}+v_{glk}=v_{glc\_uptake}$, $\mu_{atp}$was associated with $v_{atp}\geq v_{atp\_main}$ , and $\mu_{biomass}$was associated with $v_{biomass}\geq v_{biomass}^{\text{target}}$. Now left the last constraint (a),

$\text{v}_{\text{j}}^{\min}\cdot\text{y}_{\text{j}}\leq\text{v}_{\text{j}}\leq\text{v}_{\text{j}}^{\max}\cdot\text{y}_{\text{j}}$, $j\in M$ (a)

M was the set of all the reactions. They should be assigned with auxiliary variables, for *v*_j_ was the variables of the inner problem, although *y*_j_ could be regarded as parameters. Rewrited (a) as

$\text{v}_{\text{j}}\leq\text{v}_{\text{j}}^{\max}\cdot\text{y}_{\text{j}}$, $-\text{v}_{\text{j}}\leq-\text{v}_{\text{j}}^{\min}\cdot\text{y}_{\text{j}}$ , $j\in M$ (b)

It seemed OptKnock assigned $\mu_{j}$ ($j\in M$) as auxiliary variables to those constraints (b).

From the dual theory of linear programming (LP), the dual problem (DP) takes the following form (III) and *y* is the auxiliary variable vector. All the auxiliary variables, i.e. *y*, should appear in the objective function of the dual.


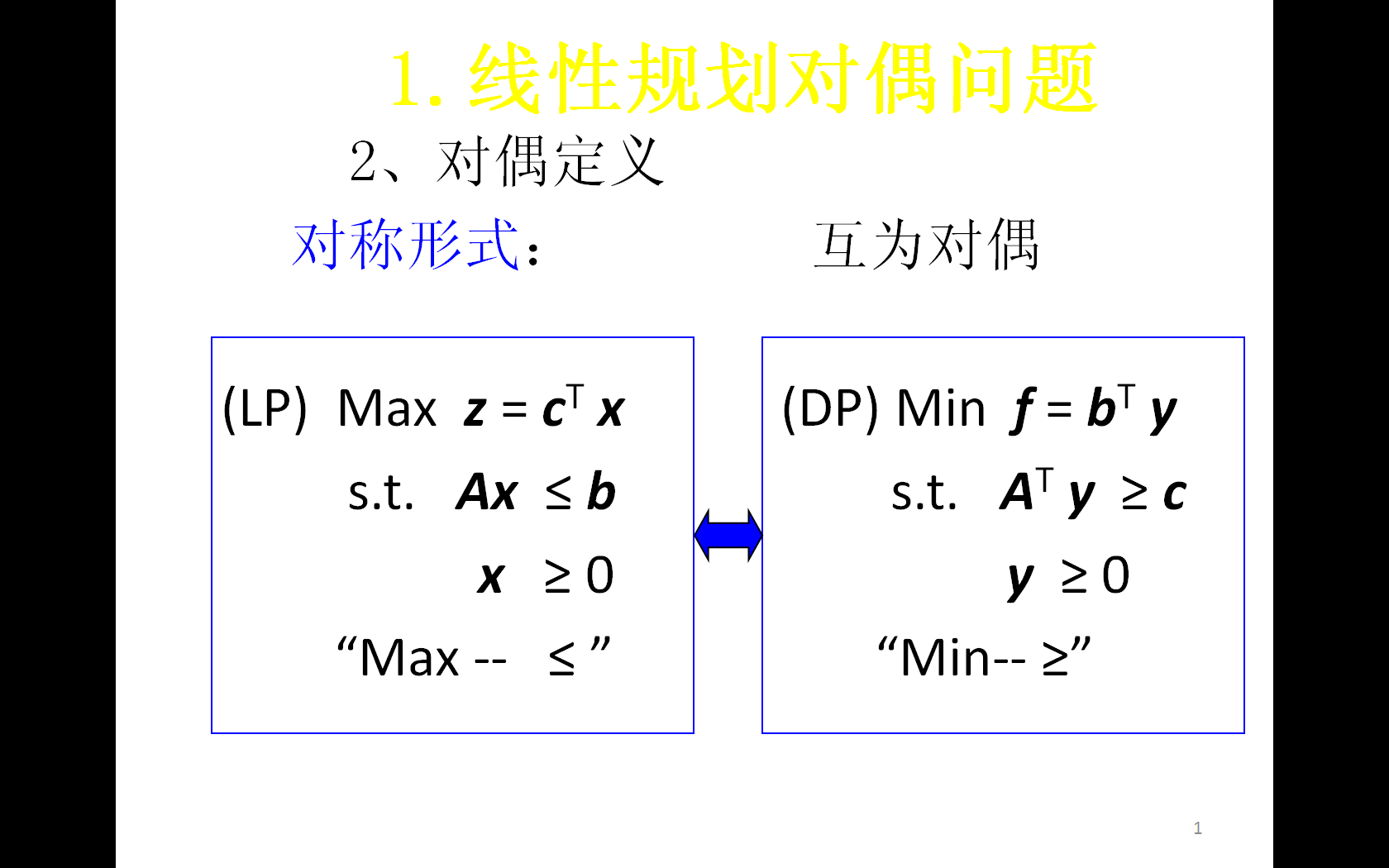
 (III)

The objective function of the dual of OptKnock was

$v_{atp\_main}\cdot\mu_{atp}+v_{biomass}^{\text{target}}\cdot\mu_{biomass}+v_{glc\_uptake}\cdot glc$ (c)

It has included auxiliary variables $\mu_{atp}$, $\mu_{biomass}$, and *glc*, while $\lambda_{\text{i}}^{\text{stoich}}$($i\in N$) was not included, for the right side of i-th stoichiometric constraint was zero. But the objective function did not include $\mu_{j}$ ($j\in M$). If we add $\mu_{j}$ to the objective function of the dual of OptKnock, the objective function should take the following form

$\begin{matrix} v_{atp\_main}\cdot\mu_{atp}+v_{biomass}^{\text{target}}\cdot\mu_{biomass}+v_{glc\_uptake}\cdot glc \\ \begin{matrix} & & \end{matrix}+\sum_{\text{j}} \text{v}_{\text{j}}^{\max}\cdot\text{y}_{\text{j}}\cdot\mu_{j}-\sum_{\text{j}} \text{v}_{\text{j}}^{\min}\cdot\text{y}_{\text{j}}\cdot\mu_{j} \end{matrix}$ (d)

Especially, (d) will appear in the final transformation for the MIBLP and be regarded as a constraint (e).

$\begin{matrix} v_{atp\_main}\cdot\mu_{atp}+v_{biomass}^{\text{target}}\cdot\mu_{biomass}+v_{glc\_uptake}\cdot glc \\ \begin{matrix} & \end{matrix}+\sum_{\text{j}} \text{v}_{\text{j}}^{\max}\cdot\text{y}_{\text{j}}\cdot\mu_{j}-\sum_{\text{j}} \text{v}_{\text{j}}^{\min}\cdot\text{y}_{\text{j}}\cdot\mu_{j}\text{＝}v_{biomass} \end{matrix}$ (e)

For (d) includes the product of the auxiliary variable $\mu_{j}$multiplying the control variable *y*_j_, thus (e) is nonlinear.

So the correct objective function of the dual for the inner problem in OptKnock should be (d) but not (c) and when it is included in the final transformation for MIBLP, the constraint (e) is nonlinear. The final transformation for MIBLP will not be a Mixed Integer Programming (MILP) but a nonlinear programming. Here we do not deny dual theory is also useful in this kind of transformation, but OptKnock did not explain how to get the final transformation as a MILP.
