# Supplementary material for: ReacKnock: Identifying Reaction Deletion Strategies for Microbial Strain Optimization Based on Genome-Scale Metabolic Network
Source: PLoS One. 2013 Dec 11;8(12):e72150. doi: 10.1371/journal.pone.0072150 (PMC3859475; doi:10.1371/journal.pone.0072150)
Supplement: Table S4 — Comparison between new predictions of OptKnock with default setting of max computation time in COBRA toolbox and old predictions (first time computation) of OptKnock with setting computation time to be 3600 s. (DOCX) [file pone.0072150.s006.docx]

Table S6. Comparison between new predictions of OptKnock with default setting of max computation time in COBRA toolbox and old predictions (first time computation) of OptKnock with setting computation time to be 3600s. Succinate, Acetate, Formate and Fumarate were improved but not great when extending the computation time, while other chemicals remained no improvement. OptKnock was instable in the case of Hydrogen production. we also provided the result of OptKnock with setting computation time as 10 minutes.

| Chemical target | Strain | Prod. rate | Growth rate | FVA max prod. rate | Reaction to be deleted as example | Computing time, min |
| --- | --- | --- | --- | --- | --- | --- |
| Succinate | (Max_Yield) | 14.93 |  |  |  |  |
|  | Wild_type | 0 | 0.885 | 0.0001 | -- |  |
|  | OptKnock1 | 0.85 | 0.81 | 0.85 | CGLYtex; FUM; IPDPS; M1PD; NTP1 | 10 min |
|  | OptKnock2 | 6.3 | 0.552 | 6.31 | ACt2rpp; GND; PSP_L; SUCDi; SUCOAS | 60 min |
|  | OptKnock3 | 9.9 | 0.117 | 9.97 | ACt2rpp; ATPS4rpp; CO2tex; PGL; THD2pp | 2days 8h |
| Ethanol | (Max_Yield) | 18.56 |  |  |  |  |
|  | Wild_type | 0 | 0.885 | 0.000024 | -- |  |
|  | OptKnock1 | 17.3 | 0.143 | 17.3 | ALAALAtex; ATPS4rpp; NDPK3; PTAr; SUCOAS | 10 min |
|  | OptKnock2 | 18.2 | 0.121 | 18.19 | ACt2rpp; ATPS4rpp; GLUDy; PPKr; SUCDi | 59 min |
|  | OptKnock3 | 18.4 | 0.104 | 18.46 | ATPS4rpp; FORtex; GLUDy; O2tex; THD2pp | 2days 8h |
| Acetate | (Max_Yield) | 25.69 |  |  |  |  |
|  | Wild_type | 1.68 | 0.885 | 1.68 | -- |  |
|  | OptKnock1 | 17.8 | 0.121 | 17.8 | ATPS4rpp; FORt2pp; LACZ; PGM; TYRtex | 10 min |
|  | OptKnock2 | 18.3 | 0.116 | 18.25 | 3OAS120; ATPS4rpp; ENO; GLU5K; SUCDi | 62 min |
|  | OptKnock3 | 23.1 | 0.119 | 23.15 | ATPS4rpp; ECOAH5; EDA; LYSt3pp; TPI | 2days 8h |
| Hydrogen | (Max_Yield) | 76.64 |  |  |  |  |
|  | Wild_type | 9.81 | 0.885 | 9.81 | -- |  |
|  | OptKnock1 | 1000 | 0.885 | 9.81 | ASPt2pp; HMPK1; ILEt2rpp; OMMBLHX3; SERTRS2 | <1 min |
|  | OptKnock2 | 1000 | 0.885 | 9.81 | no deletion | <1 min |
|  | OptKnock3 | 1000 | 0.885 | 9.81 | ASPt2pp; HMPK1; ILEt2rpp; OMMBLHX3; SERTRS2 | <1 min |
| Formate | (Max_Yield) | 43.69 |  |  |  |  |
|  | Wild_type | 0.0021 | 0.885 | 0.00223 | -- |  |
|  | OptKnock1 | 24.9 | 0.139 | 24.9 | DRPA; GLCt2pp; H2Otex; PGM; PPC | 10 min |
|  | OptKnock2 | 25.5 | 0.142 | 25.54 | 12PPDStex; H2Otex; PGI; PGL; PGM | 61 min |
|  | OptKnock3 | 32.08 | 0.127 | 32.087 | ACALD; EDA; H2Otpp; PGM; PPS | 2days 8h |
| Glycolate | (Max_Yield) | 25.69 |  |  |  |  |
|  | Wild_type | 0 | 0.885 | 0.000039 | -- |  |
|  | OptKnock1 | 17.9 | 0.132 | 17.9 | ACt2rpp; ATPS4rpp; CO2tex; G3PT; PPKr | 10 min |
|  | OptKnock2 | 17.4 | 0.142 | 17.43 | ACtex; AKGDH; ATPS4rpp; FALDtpp; GLCNtex | 60 min |
|  | OptKnock3 | 17.9 | 0.132 | 17.98 | ACt2rpp; ATPS4rpp; CO2tex; G3PT; PPKr | 2days 8h |
| D-Lactate | (Max_Yield) | 18.56 |  |  |  |  |
|  | Wild_type | 0 | 0.885 | 0.000019 | -- |  |
|  | OptKnock1 | 11.8 | 0.137 | 11.81 | ATPS4rpp; DAGK120; O2tex; PTAr; RPE | 10 min |
|  | OptKnock2 | 18.5 | 0.101 | 18.51 | ATPS4rpp; ETOHt2rpp; IMPD; LEUtex; O2tex | 60 min |
|  | OptKnock3 | 18.5 | 0.101 | 18.50 | ATPS4rpp; ETOHt2rpp; O2tex; PROD2; SUCOAS | 2days 8h |
| Fumarate | (Max_Yield) | 16.08 |  |  |  |  |
|  | Wild_type | 0 | 0.885 | 0.0000082 | -- |  |
|  | OptKnock1 | 8.38 | 0.223 | 8.39 | CO2tex; FUM; GLCDpp; NADH16pp; PGK | 10 min |
|  | OptKnock2 | 9.4 | 0.127 | 9.39 | 3HAD140; ATPS4rpp; CO2tpp; PFL; TKT2 | 60 min |
|  | OptKnock3 | 12.7 | 0.216 | 12.78 | CO2tex; GLUDy; PFL; PYRt2rpp; TPI | 2days 8h |
| Threonine | (Max_Yield) | 11.22 |  |  |  |  |
|  | Wild_type | 0 | 0.885 | 0 | -- |  |
|  | OptKnock1 | 0 | 0.885 | 0 | AOXSr; DM_AACALD; FE3tex; HACD8i; LCADi | 10 min |
|  | OptKnock2 | 0.000000012 | 0.774 | 0.0000237 | ACALDtpp; ACtex; ETOHt2rpp; Htex; TRPS1 | 60 min |
|  | OptKnock3 | 0.0000093 | 0.802 | 0.0000092 | G5SD; Htex; R15BPK; RNDR3b; THRD | 2days 8h |

OptKnock1: maximal computing time is set to 600 sec.

OptKnock2: maximal computing time is set to 3600 sec.

OptKnock3: default setting of max computation time in COBRA toolbox.

The following constraints were applied: glucose consumption rate is 10, cell growth is no less than 0.1, maintenance energy metabolism is 8.39, oxygen consumption rate is no higher than 18.5. All the rate unit is mmol/g(Dw)h. Max_yeild means the theoretical conversion ratio at the given condition.
